# Experiences and Beliefs on Tobacco Use, Cessation in India: A Qualitative Study

**DOI:** 10.5334/gh.1267

**Published:** 2023-09-22

**Authors:** Aswathy Sreedevi, Anindo Majumdar, Yvonne Olando, Marie Chan Sun, Catriona Jennings, Kemi Tibazarwa, Holly Gray, Katarzyna Zatonska, Rinu PK, Shana Shirin Najeeb

**Affiliations:** 1 Amrita Institute of Medical Sciences, Kochi, Kerala, IN; 2 Dept of Community Medicine, All India institute of Medical Sciences, Bhopal(AIIMS), Bhopal, IN; 3 National Authority for the Campaign against Alcohol and Drugs Abuse (NACADA), Nairobi, KE; 4 Department of Medicine, Faculty of Medicine and Health Sciences, University of Mauritius, Reduit, MU; 5 National Institute for Prevention and Cardiovascular Health, Univerity of Galway, IE; 6 Muhimbili National Hospital, Dares salam, TZ; 7 Robin Martin, London, UK; 8 Dept Of Social Medicine, Medical University, Wroclaw, PL; 9 John Snow India Private Limited, IN; 10 Dept Of Community Medicine, AIMS, Kochi(Former), IN

**Keywords:** Tobacco use, Health care facility, experience, cessation, India, Qualitative

## Abstract

**Background:**

Almost 80% of global tobacco usage is concentrated in low- and-middle-income countries (LMICs) like India. Added to this, there is dearth of dedicated tobacco cessation specialist services in healthcare settings in these regions. Identification of challenges in the delivery of cessation interventions and understanding the experiences of tobacco users will aid in formulating successful quit strategies.

**Objectives:**

This qualitative study in India aimed to understand the perspectives of tobacco-using patients in healthcare facilities regarding tobacco use and cessation.

**Methods:**

This qualitative study was conducted in urban and rural areas of four study sites, two each in the North and South India. Using purposive sampling, patients who were tobacco users were selected from healthcare facilities. The interviews were transcribed, coded and organised into themes. Analysis was done using NVivo 10 software.

**Results:**

A total of 22 in-depth interviews were conducted on participants aged 23 to 80 years who were either current or past tobacco users. A majority of the participants were aware of their increased health risks associated with tobacco consumption and had attempted quitting; however, barriers such as peer influence, formed habit, certain cultural barriers and the addictive nature of nicotine prevented them from successfully quitting. Familial and peer support, the government’s role in spreading public awareness, and limiting the sale of tobacco were stated as facilitators for tobacco cessation.

**Conclusions:**

The findings of this study point out that despite awareness of the perils of tobacco among smokers, there are various barriers and beliefs related to tobacco use and cessation. These findings would prove advantageous for policy-makers to implement and promote addiction treatment programmes for successful tobacco cessation efforts. In order to optimise strategies, policies must be well informed by ongoing dialogue between the public, service providers and policy-makers.

## Introduction

Tobacco and tobacco products are known to be highly addictive substances due to their nicotine content, and their use is a common risk factor for the four main groups of non-communicable diseases (NCD), namely cardiovascular disease, chronic lung disease, cancer and diabetes [1]. Usage of tobacco is the single largest preventable cause of NCDs [2]. According to the World Health Organization (WHO), more than eight million people die annually from tobacco use globally [3]. Almost 80% of these deaths occur in low- and middle-income countries (LMICs) [4], the areas principally targeted by tobacco industry marketing [3].

It is therefore imperative to accelerate and strengthen tobacco cessation support in LMICs through effective tobacco cessation programmes. The cost-effectiveness of tobacco cessation programmes has been well documented [5,6]. There is an urgent need to provide support at an individual level for quitting tobacco, especially in LMICs. This is reflected in Article 14 of the Global Framework Convention on Tobacco Control (FCTC), which focuses on demand reduction measures for tobacco cessation, as well as in the WHO MPOWER initiative’s ‘O’ for offering help to quit [7,8].

India is the second-largest producer of tobacco and is third in the consumption of tobacco [9]. A large variety of tobacco products are available at low prices in the country [10]. Around 267 million adults (aged 15 years and above) in India are tobacco users, according to the latest Global Adult Tobacco Survey [11]. India has enacted several comprehensive tobacco control measures over the years, such as the 2003 Cigarette and Other Tobacco Products Act (COTPA) legislation, and was also one of the forerunners to ratify the WHO FCTC in 2004 [12]. The Ministry of Health and Family Welfare in India issued tobacco treatment guidelines in 2011 which recommend that physicians in primary care and other settings identify and treat every tobacco user seen in healthcare settings. These guidelines specify that all healthcare providers must provide 5As—‘Ask’, ‘Advise’, ‘Assess, ‘Assist’ and ‘Arrange’—for tobacco cessation counselling as a part of routine healthcare consultations. Dedicated tobacco cessation specialist services should also be set up in healthcare settings [13].

There is currently a substantial gap between the evidence base available and its implementation. For example, it has been shown that the majority of the patients visiting primary healthcare facilities in India are neither receiving tobacco cessation counselling nor being referred to tobacco cessation centres [14]. A recent study also reported that concordance rates between physicians and patients for various components of 5A’s varied from 7.4% for ‘Advice’ to 56.4% for ‘Arrange’ [15]. These findings demonstrate the wide disparities that exist in terms of what happens in routine clinical practice and what is perceived by patients and healthcare professionals as actions taken towards tobacco cessation.

There is a dearth of qualitative data on implementation of tobacco cessation interventions in healthcare facilities in LMICs. This issue has to be addressed urgently, given the lack of implementation of cessation support services in a diverse country like India. The objective of this qualitative study was to understand the perspectives of tobacco-using patients in healthcare facilities regarding tobacco use and cessation.

## Methodology

This qualitative study was carried out after obtaining clearance from the independent ethics committee of the Centre for Chronic Disease Control, New Delhi, India (no. CCDC_IEC_11_2016 dated 31/01/2017) and was conducted as part of a larger multi-country study (India and Kenya). It involved finding out the barriers and facilitators of tobacco cessation interventions, which included data collection from patients, healthcare providers, programme managers, healthcare providers and policy-makers. Using purposive sampling, two study sites were selected, one in North India and one in South India. The country’s capital, New Delhi, and Kurukshetra district, in the state of Haryana, were selected from North India. Two densely populated districts from Kerala were chosen from South India, namely the Ernakulam and Thiruvananthapuram districts. As India is a large country with substantial geographic and sociocultural differences, the selection of the study sites was carried out based on feasibility. It must be noted that under the federal structure in Haryana and Kerala, health is largely the state’s responsibility; whereas in New Delhi, which is the capital city and a Union Territory, it is more under central government control. Both rural and urban areas from these four sites were selected.

The participants were selected using a purposive sampling technique, depending on local requirements. Purposive sampling for patients was done based on characteristics such as age, gender, residence (i.e., North India or South India), urban or rural, varying levels of socioeconomic status (SES), the level and type of healthcare facility the patient usually receives care from (primary/secondary/tertiary and public/private), tobacco use status (current tobacco user/past tobacco user and smoker/smokeless tobacco user) and tobacco intervention status (those currently under pharmacological or non-pharmacological intervention/those who received and successfully completed interventions previously/those who started pharmacological or non-pharmacological interventions and did not complete them). Patients were interviewed until data saturation was reached, which determined the sample size (i.e., 22 in-depth interviews [IDIs]). All the selected participants who were approached for an IDI had agreed to the interviews and gave consent.

### Operational definitions

A ‘current tobacco user’ was defined as someone who is presently using any form of tobacco or has used it within the last month. A ‘past tobacco user’ was defined as someone who has not used tobacco for more than a month. The healthcare professionals were physicians, psychologists, nurses, community health workers, pharmacists, social service professionals, counsellors and other allied healthcare professionals based in or affiliated with healthcare facilities involved in or likely to be involved in delivery of cessation interventions.

Before interviewing the patients, permission from relevant authorities and the hospital administration was obtained. Among the government-funded health facilities, the primary healthcare facilities included the Primary Health Centres (PHCs) and the Community Health Centres (CHCs), secondary healthcare facilities included the District Hospitals and the tertiary healthcare facilities involved big hospitals associated with medical schools. In the private healthcare system, the single-physician clinics and multi-speciality clinics or polyclinics with only outpatient care (no inpatient care) were considered as primary care facilities and those with inpatient care (such as big hospitals) as tertiary care facilities.

Using purposive sampling, patients were selected by the researcher from healthcare facilities according to their background characteristics, which were obtained following discussions with their consulting physicians based on the records maintained. This was done on each day of the visits by data collectors to specific outpatient departments. Appointments for the interviews with participants were taken through phone calls or personal visits. After obtaining the written informed consent, participants were interviewed with the help of interview topic guides at a location which was considered convenient by the patient, either at the healthcare facility or at the patient’s home. The interviews were recorded and conducted either in English or in the local language, as per the participant’s choice. The average duration of the interviews was 24 minutes. Of the 22 participants, 12 participants were from South India and 10 from North India.

Prior to the conduct of the study, interview schedules were developed and translated into the local language after extensive formative research. Face-to-face interviews were conducted by investigators and staff trained in conducting qualitative interviews. All interviews were conducted by RPK and AM. AM is trained and experienced in qualitative methods and analysis, and he trained RPK in data collection for about two sessions of four hours each, especially in conducting good quality IDI. AM and AS coordinated the data collection in India. The interviews were recorded using a digital voice recorder. Field notes were taken in a diary and covered a wide range of observations specific to the context.

The audio-recorded data was later transcribed verbatim and translated into English, where applicable, by RPK. Some of the transcripts were returned to participants for comment and correction, to check whether their interviews were correctly interpreted. However, this step was only done in 9 out of 22 IDIs due to lack of sufficient resources, time and refusal by some of the participants. The transcripts were then coded and organised into themes and sub-themes. Analysis was carried out with the assistance of QSR International NVivo 10 qualitative analysis software.

Two data coders coded the data. Coding were done separately by RPK and AM. In case of disagreement, a final coding sheet was developed in discussion with another member of the team, primarily AS. Finally, all the authors validated the themes which emerged and the quotes included. Inductive analysis was done through reading and rereading of the interview transcripts through an iterative process, and thus codes and sub-codes were generated. From the resultant codes and sub-codes, categories were developed by grouping codes which were highlighting a similar issue. The resulting categories were then merged and grouped as per similarities in terms of emerging themes and sub-themes. Verbatim quotes have been mentioned under relevant themes and sub-themes. The description of the Coding tree is provided as annexure 2.

The reflexivity was addressed in the following manner. The study was coordinated in India mainly by the first two authors AS and AM. Data collection was mainly conducted by RPK, who was trained by AM. Some of the interviews were also conducted by AM. To avoid bias, the interviews conducted by AM were in Kerala, where he had never worked or resided before. Similarly, the interviews conducted by RPK were conducted in the health facilities of Haryana and Delhi, where she had not previously worked, being a resident of Kerala. Although RPK has a BSc (Nursing) and MPH and did collect some data in Kerala, she had never worked in Kerala. Although AS has worked in Kerala and is a resident of Kerala, AS was not involved in data collection per se but helped in coordination of the process. Manuscript writing, editing and review by other authors of the team who are from countries other than India helped in decreasing the subjective bias in analysis by the Indian authors.

The first two authors are medical professionals with a three-year postgraduate specialisation in community medicine. The other investigators with qualitative research experience supported the implementation of the project. The main data collector, RPK, was a nurse. Since all three were primarily healthcare providers, this may have affected their interactions with patients and influenced their interpretations of the responses.

## Results

The age of the study participants ranged from 23 years to 80 years. Participants were included from urban and rural areas of New Delhi and the Ernakulam, Trivandrum and Kurukshetra districts in India (Table 1). Participants were either current or past smokers or users of smokeless tobacco. Both female and male participants from each category were included.

**Table 1:** Distribution of respondents according to sociodemographics (*N* = 22).


S.NO.	VARIABLES	CATEGORY	FREQUENCY	PERCENTAGE

1.	Gender	Male	15	68.2

		Female	7	31.8

2.	Residence	Urban	9	40.9

		Rural	13	59.1

3.	Socioeconomic status	High	10	45.5

		Low	12	54.5

5.	Healthcare facility providing treatment	Primary	13	59.1

		Secondary	4	18.2

		Tertiary	5	22.7

6.	Tobacco product used	Cigarettes/Bidi	17	77.3

		Smokeless tobacco	5	22.7

7.	Age	60 years and above	07	31.8

		Below 60 years	15	68.2


Six themes emerged from the study: (1) perceptions regarding tobacco use, (2) tobacco use and experiences, (3) barriers to cessation and challenges in quitting smoking, (4) facilitators of tobacco cessation, (5) the roles played by government and society in tobacco cessation and (6) curbing tobacco use.

### 1. Perceptions regarding tobacco use

#### 1. Harmful nature of tobacco products and high addictive potential

The principal theme which emerges from this qualitative study is the awareness of participants on the fact that tobacco use is harmful and detrimental to health. Television advertisements, health warnings on tobacco packaging and advice from healthcare providers were cited as sources of information by a majority of users.

Because it is written in each packet that it’s harmful for health, everybody knows about the side effects. But it becomes a habit that they aren’t able to quit … and as all know, habits are difficult to leave. (30-year-old male, current smoker, urban area)

When asked about the various ill effects of tobacco, participants responded that tobacco use may predispose to cancers, including lung, gastrointestinal and oral cancers; respiratory and cardiovascular problems; and oral health problems. This qualitative study also revealed the awareness of participants on chronic pain in gingiva, raised blood pressure and stroke.

Smoking increases the probability of getting cancer. Mm … and obviously lung capacity decreases. (25-year-old female, current smoker, urban area)

If a person is smoking, then that smoke is causing harm to somebody who is standing next to him also. I smoke in areas where nobody is present at that time. (57-year-old male, smoker, rural area)

In addition to reporting on the awareness of participants on the ill effects of tobacco, this study also revealed obliviousness to the harmful effects of tobacco use.

I don’t have any opinion about it. When I am free, I just keep the tobacco inside my mouth, and I feel good about it. They say it can cause cancer and all. But I never believed them. Because even young people who don’t use this are also dying of cancer right? (72-year-old female, current smokeless tobacco user, rural area)

See all tobacco users won’t get cancer! There are people in my place who are 90 to 95 years, and they still use tobacco. But they are still healthy, and they don’t have any issues. (78-year-old male, past smoker, rural area)

### 2. Tobacco use and experiences

#### 2a. Withdrawal: Side effects of tobacco usage experienced by participants

The reported side effects experienced by participants included anger issues, headache, memory loss, laziness, sexual problems and difficulty in eating and drinking.

I get angry and have [a] headache. If I don’t get it (tobacco) for a long time, then I scold my children or grandchildren unnecessarily. (80-year-old female, current smokeless tobacco user, rural area)

The benefits of quitting smoking, such as better sleep and better appetite, were reported.

I realised that my memory loss and laziness which I was experiencing at that time may be due to my tobacco use. I had problems in my sex life too. (40-year-old male, past smoker, urban area)

After quitting smoking, I can have food properly now. There was a time when I couldn’t sleep, even if I smoke a packet of beedi. Now I have better sleep. (78-year-old male, past smoker, rural area)

#### 2b. Reasons for continued use of tobacco

The majority of participants stated that they started smoking due to curiosity and social influence. However, they later realised it was highly addictive and found it difficult to quit, as it had become a habit.

I tell them that, okay, I will stop. Then when I come home, I chew tobacco (laughing). I know that they are telling for my benefit. But when I sit alone, I feel like having it. And I just chew it … no reason as such. (72-year-old female, current smokeless tobacco user, rural area)

The majority of participants expressed that they experience the urge to smoke when they are alone or sitting idle. Some of the participants said that they smoke when they have pain or a toothache, as it helps them forget the pain. Some used it to avoid falling asleep during nightshifts.

### 3. Barriers to cessation and challenges in quitting smoking

The primary barrier to quitting smoking was their long-standing dependence on tobacco since childhood and a reported lack of self-efficacy.

What to say. … I want to quit, but I am not able to. When I go to [the] hospital, they tell me to quit. But I am unable to. (60-year-old female, current smokeless tobacco user, rural area)

Culture-specific perceptions were also mentioned as a barrier to smoking cessation.

My daughter-in-law also takes it. Among Jats [a community in North India], it’s common [practice]. (78-year-old female, smoker, rural area)

Public stigma and gendered bias to smoking were also put forward as barriers to smoking cessation.

With women there is also an added thing. Like [if you smoke], you must be too modern, etc., the kind of language people use! There are questions about smoking in the patient intake which are not even asked to women. Since the doctors or nurses presume that women don’t smoke. (36-year-old female, smoker, urban area)

### 4. Facilitators of tobacco cessation

#### 4a. Factors that aid successful quitting and harm reduction

Intrinsic motivation and indomitable will power were reported as the key facilitators to quitting tobacco use, even if users had to take medicines to aid the process.

Self-control is the main thing. (57-year-old male, smoker, rural area)

The occurrence of health problems was reported as a cue to action for smoking cessation.

My BP rose. it was nearly 180/110. [The] doctor told me smoking is the main cause and [I had] better stop using it. So that was that, and I left it. (36-year-old male, past smoker, urban area)

When I attended one of the cessation classes in the clinic, they told [us that] if nicotine content is increased, then it can lead to psychiatric problems. Then I decided to quit. (36-year-old, past smoker, urban area)

Social image and social acceptance were also reported as encouragement to stop using tobacco. Financial issues also motivated participants to quit.

I am a social person. I want to mingle with everyone and don’t want to be avoided. Also, I have two daughters. If I don’t quit now, then it will affect their future also. So I thought, I spent my 40 years like this, so coming years I want to live it in a better way. (40-year-old male, past smoker, urban area)

When I started smoking, the price of one beedi was 25 paisa; now its 15 rupees. I pondered over it. I need 3 packets of beedi for one day, for which I need to spend 45 rupees; but for one tea, it will cost only 10 rupees. Thus I decided not to smoke. … I managed because I didn’t have money for it. (78-year-old male, past smoker, rural area)

#### 4b. Practices adopted by participants to quit smoking

Participants who were ex-smokers said that they consumed home remedies such as cardamom, rice water, coconut water and mango leaf to help in quitting tobacco. They also made sure that they were never alone and kept themselves engaged, which helped them in quitting.

Whenever I had the urge, I used to eat cardamom. Also, I tried to make sure that I never sit alone. Spending time with kids helped me a lot. … Drinking rice water and tender coconut water provides a freshness to the mind and avoids drying of mouth and throat. I used to have 10 pan per day. That I reduced to 6, 4, and 2 per day. Then in between I chew cardamom. I started drinking hot water. After stopping, thirst will increase, so I had more water. (40-year-old male, past smoker, urban area)

### 5. The roles played by government and society in tobacco cessation

#### 5a. The role played by government in tobacco cessation

Participants had mixed views regarding the role that the government should play in tobacco cessation. It was reported that the government should stop sales of tobacco at several places. It was also felt that the government needs to play a more active role in tobacco cessation and seek the collaboration of private players such as private hospitals, clinics and non-governmental organisations (NGOs).

There is a limitation to what government [does]; it is not like private firm. Private firms aim for profit. Government organisations don’t make much income. (50-year-old male, past smokeless tobacco user, rural area)

Fund for arranging the classes and other programmes. Mainly they [government] are providing the fund. (23-year-old male, smoker, rural area)

#### 5b. The role played by society in tobacco cessation

It was unanimously agreed that family support helped smokers to quit.

Family support is very important. My family didn’t know that I am smoker for 10 long years. In such cases, family support can’t do much. Thus, he needs external support or his friend’s support. (34-year-old male, current smoker, urban area)

My family asked me to get treatment for it. I had lots of pressure from home to stop tobacco. They helped me. (36-year-old male, past smoker, urban area)

My children were unhappy about me smoking. So, I thought of quitting. …. Hmm … but still I couldn’t quit. (64-year-old female, current smoker, rural area)

### 6. Curbing tobacco use

#### 6a. At the individual level

Participants put forward that distancing themselves from public places or social gatherings where people smoke helped them to stay smoke-free.

It is a question of finding something [to distract] at the times I usually smoke. … Say, I would go for a walk or eat a piece of chocolate. … If you are going for a dinner or are in an environment where other people are smoking, then of course there will be temptation. So don’t go to such places. (25-year-old female, current smoker, urban area)

#### 6b. At the government level

The general view shared by participants was the need for a complete ban of tobacco products—both the production and sale.

I do think that tobacco should be effectively illegalised, because if you really want to solve the problem, all you need to do is stop the supply. If tobacco is bad for you, then, why the government is enabling tobacco production? It’s really as simple as that. … The government plays a huge role. From the policy or advocacy point of view, make it difficult for the tobacco business (to grow). It should be across the chain. If it is that easy for some states to ban alcohol supply, it should be easy for states to stop tobacco. (25-year-old female, current smoker, urban area)

They [government] want money and at the same time talk about tobacco cessation. Government can easily ban tobacco if they want to. (78-year-old male, past smoker, rural area)

People felt that newspaper advertisements from the local self-government regarding counselling and cessation centres in each area would increase awareness regarding treatments for tobacco cessation. Increasing taxes on tobacco products was perceived as being a good alternative. More funding for the healthcare system and for tobacco cessation support groups was also put forward.

So, if the government programmes aim to reduce the number of tobacco users, then they have to create motivational things for people to not use tobacco. (36-year-old female, current smoker, urban area)

#### 6c. At the community level

Participants stressed the need to generate more awareness and reinforce health warnings as one of the most important interventions, especially regarding the harmful effects of tobacco use, including its severity on media.

It was suggested that people and organisations outside the health system can also contribute in generating awareness. Schools were seen as a source of awareness for students and family members. Although, ‘no smoking’ rules in workplaces was said to be important, no specific suggestions were given, citing reasons of interfering with personal rights.

Children in my home used to tell. They get information from their schools. They came and told me that smoking can cause diseases. (78-year-old female, current smoker, rural area)

Actually, schools and workplace[s] can play [a] greater role than [the] healthcare system. I would find useful if it is in a class ten or class nine notebook; you put in a section on tobacco, what and why tobacco is harmful to human body. (25-year-old female, current smoker, urban area)

## Discussion

This qualitative study revealed the awareness of participants on increased health risks associated with tobacco consumption and highlighted the barriers to quitting, which include peer influence, cultural context and, most importantly, tobacco addiction.

Smoking cessation, as a prime intervention to reduce the risk of developing NCDs is indeed a challenging process [16] which in general requires medication and/or behavioural support. Literature is replete with evidence on the efficacy of prescribed medications with respect to achieving long-term abstinence from tobacco use [17]. Behavioural support, in the form of counselling or financial incentives, has also been shown to increase the chances of successful tobacco quitting [18]. A systematic review by Stead et al. found that nicotine replacement therapy (NRT) increases success quit rates by 50% to 70%, irrespective of the setting [19], while other reviews show that the combination use of NRT is more effective than single type of NRT [20]. Indeed, when combined with behavioural support, pharmacotherapy increases tobacco cessation rates [21]. In light of the evidence in the literature and the lack of self-efficacy reported by smokers in this study, we advocate for the training of healthcare professionals in tobacco treatment and behavioural counselling.

Lack of a healthcare system infrastructure, low political priority and lack of funding have been cited as barriers to implementing the Framework Convention on Tobacco Control Article 14 [22]. This is worsened by poor attitudes by healthcare provider towards offering tobacco dependence treatment and nicotine substitution therapy [22]. Our study, with the objective of understanding the perspectives of patients, revealed a lack of awareness about NRT. As World Heart Federation Emerging Leaders of the Tobacco Control Cohort (2016), with some of us based in developing countries, namely India, Kenya and Mauritius, we believe there is a gap in the training with respect to tobacco treatment, especially in developing countries. Hence, it is important that healthcare professionals be empowered with the required relevant training to provide smokers with treatment along with behavioural counselling.

The role of the government in curtailing the production and sale of tobacco, which includes mandatory pictorial warnings and increasing taxes on tobacco products, was viewed to be a facilitator for cessation. The roles of government and international organisations like the WHO have been well documented over the years [23]. Most recently, the government of New Zealand proposed a groundbreaking law—which can also be implemented by other countries across the globe—that aims to make a ‘smoke-free next generation’ by making it illegal for anyone who turns 14 after 2027 to buy cigarettes [24].

Participants also viewed familial and peer support as playing a huge role in cessation efforts. Social support contributes positively to initiation and cessation of tobacco use [25]. Hence, tapping into social networks can increase cessation rates. It must be noted that patients failed to mention the role of Health care providers in tobacco cessation which points to the gaps in healthcare delivery existing in the area.

Smokers experienced health issues such as decreased appetite, reduced sleep, headache and sexual problems. These findings are in line with previous articles where smoking was reported to contribute to sexual health issues such as erectile dysfunction and decreased libido [26] and disrupt normal sleep architecture [27]. Participants resorted to taking spices, such as cardamom and mango leaves, to alleviate their urge to smoke. Our finding is in line with similar studies which found that chewing confectionary gums tends to decrease nicotine withdrawal symptoms and helps individuals due to the flavour of the gum chewed [28]. From a monetary perspective, a randomised field study by Sindelar and O’Malley found that financial incentives may generate better quit rates than the health messages alone [29].

Awareness of health hazards related to tobacco use needs to be mandated in schools and colleges, which are the areas where the initiation of tobacco use often occurs. Stringent legislation banning smoking in public places is required. Although various anti-tobacco policies have been enacted to fight tobacco usage, the majority of tobacco users are dependent, making quitting a challenging task. An estimated additional 160 million global deaths may occur among tobacco users by 2050 due to a lack of cessation support [29].

Since, in the present study, patients were selected from healthcare facilities, following discussions with their consulting physicians, on the day of visit by data collectors, some bias in selection is expected by the consulting physician. Also, since the participants were patients recruited from healthcare facilities, some of the information given by them may have been biased, particularly those who were interviewed in the hospital premises itself, due to power imbalances. Also, patients are different from healthy people living in the community, as some of them might already be suffering from NCDs and be long-term or heavy users of tobacco. The information regarding NCDs was not sought in our study.

In order to increase effectiveness and optimise cessation strategies, national policies concerned with tobacco cessation must be well informed by collaboration and ongoing dialogue between the public, service providers and policy-makers. Providing tailored supportive interventions which consider individual circumstances, cultural background and health status can be fruitful in aiding quitting among tobacco users. Halting the prevailing NCD epidemic will only be possible through the collaborative efforts and advocacy work of all stakeholders in tobacco control.

## Additional File

The additional file for this article can be found as follows:

10.5334/gh.1267.s1 Supplementary Files. Code book, Tree map, Cluster Analysis and interview guide.
