# Supplementary material for: Experiences and Beliefs on Tobacco Use, Cessation in India: A Qualitative Study
Source: Glob Heart. 2023 Sep 22;18(1):51. doi: 10.5334/gh.1267 (PMC10516136; doi:10.5334/gh.1267)

Supplementary Files

Click here to access/download;Supplementary Files;Cluster Analysis Nvivo.jpg

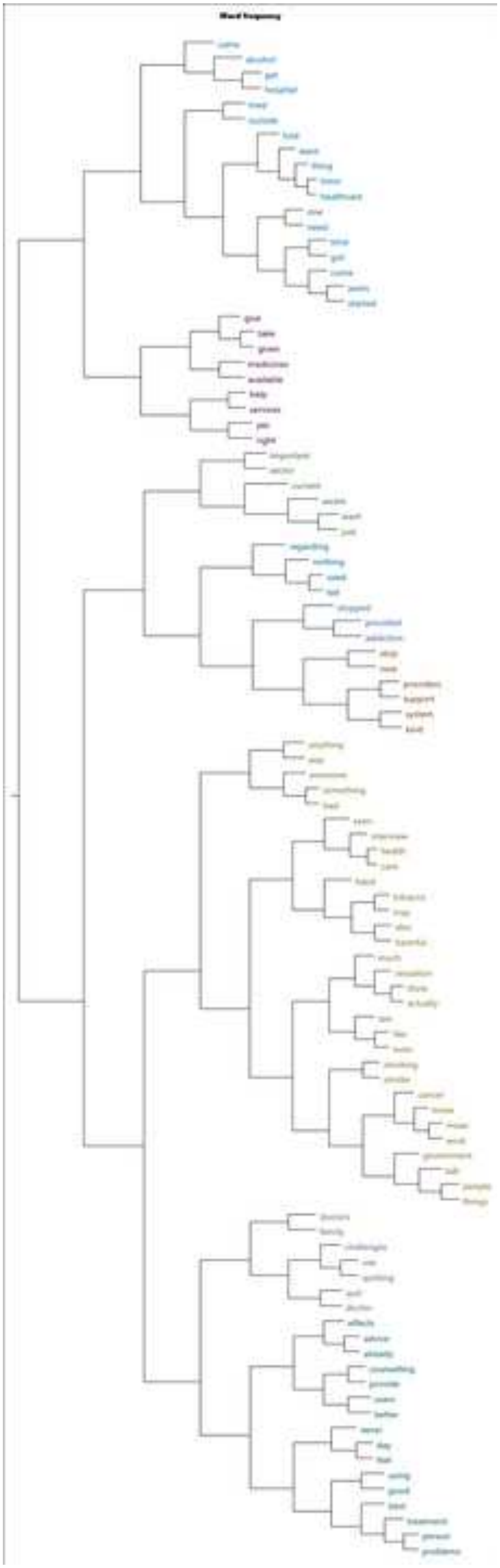

Codebook (Exported from NVivo)

Implement Cessation_Patients_India

Codes

Name	Description	Files	References
addiction among young population		1	1
Awareness regarding harmful effects of tobacco use		22	34
Challenges faced for quiiting		10	13
peer pressure		8	16
smell of tobacco		2	3
urge to use tobacco		7	10
withdrawal symptoms		3	3
changes happend after quitting		1	2
cultural implications		1	1
gender		2	3
General thoughts about tobacco use		22	31

government role in tobacco cessation		10	16
harmful affects of tobacco in patients experience		4	7
help from organizations outside the health system		15	28
motivation for quitting		5	5
money factor		2	4
poor health condition		6	7
religious support		4	5
self motivation		17	43
public smoking		3	5
quitting process		4	8
reason for tobacco use		8	14
role of family or friends in quitting		11	27
role of schools		2	3
suggestions		1	1

ban tobacco		12	19
create awareness		9	17
stop production of tobacco		2	2
stop selling tobacco in loose		1	1
taxation		3	12

Interview guide - Patients

1. What are your general thoughts about tobacco use?
2. What do you know about the harmful effects of tobacco use?
3. What types of support/treatment can a tobacco user receive for cessation?
4. Who are the persons you think can provide best advice or counselling regarding tobacco cessation?
5. In the healthcare setting where you derive most of your care, what roles do doctors or other healthcare providers play in the prevention and treatment of tobacco dependence. (Probe specifically for doctors, nurses, counsellors, pharmacists, other allied health professionals)

Questions 6-12, Ask only to a past tobacco user

6. What factors motivated you to quit tobacco use?
7. How did you manage to quit tobacco use successfully?
8. How did you decide to seek care? Did your family help in this process? Or any organization? Or your healthcare provider?
9. Who supported you in your quit process? How did you access his/her services?
10. Have you ever tried quitting tobacco use? What was the role of healthcare providers in that (Ask to a current smoker who has tried to quit at least once)?
11. How can people or organizations outside the health sector help healthcare providers for providing better tobacco cessation support?
12. How can the government provide more support to health healthcare providers for providing better tobacco cessation support?

Supplementary Files

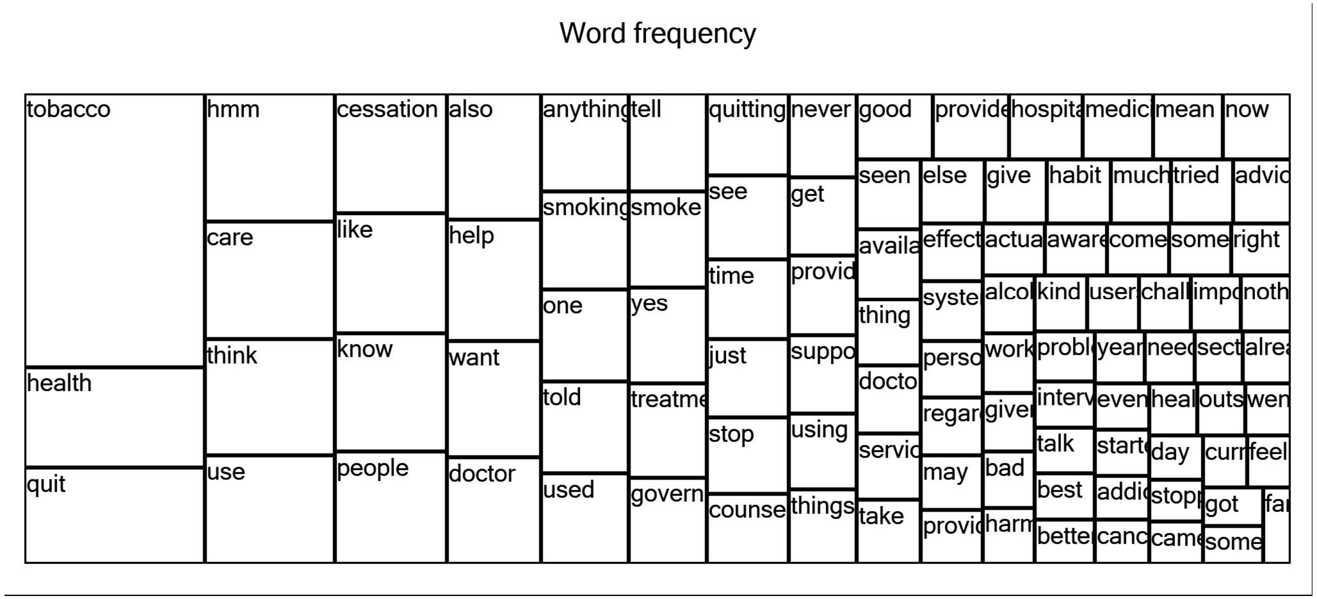

Supplementary Files
Click here to access/download;Supplementary Files;Word cloud_Nvivo.png

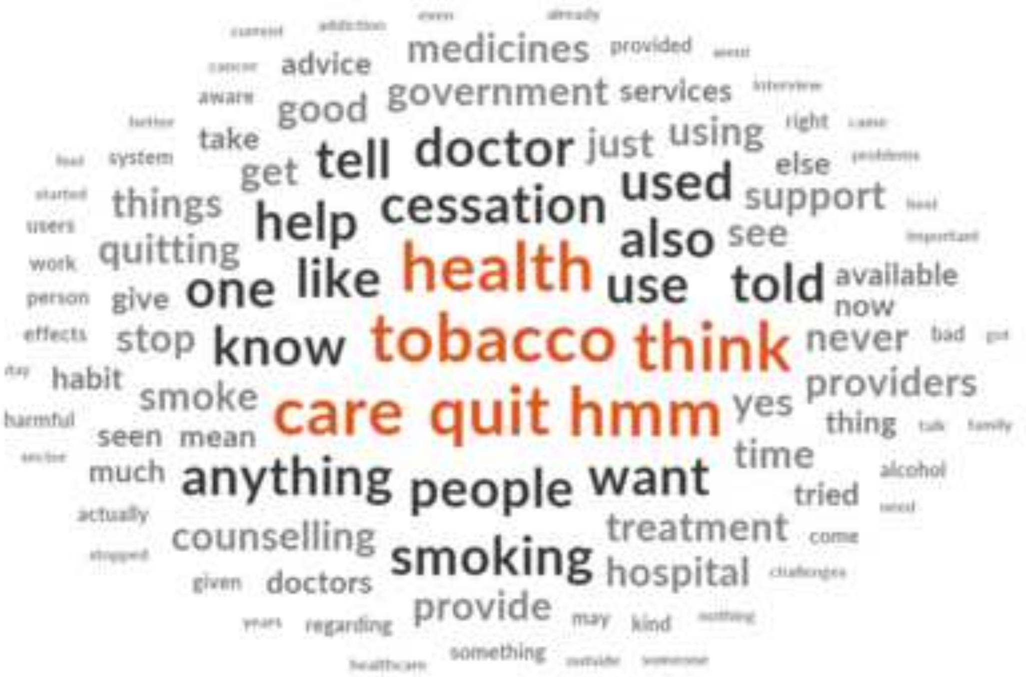

Supplement: Supplementary Files. — Code book, Tree map, Cluster Analysis and interview guide. [file gh-18-1-1267-s1.pdf]
